# Feasibility, Acceptability, and Protective Efficacy of Seasonal Malaria Chemoprevention Implementation in Nampula Province, Mozambique: Protocol for a Hybrid Effectiveness-Implementation Study

**DOI:** 10.2196/36403

**Published:** 2022-09-23

**Authors:** Kevin Baker, Pedro Aide, Craig A Bonnington, Christian Rassi, Sol Richardson, Arantxa Roca-Feltrer, Maria Rodrigues, Mercia Sitoe, Ivan Alejandro Pulido Tarquino, Sonia Enosse, Caitlin McGugan, Eva Amelia de Carvalho, Francisco Saute, Alfredo Gabriel Mayor Aparicio, Baltazar Candrinho

**Affiliations:** 1 Department of Public Health Sciences Karolinska Institute Stockholm Sweden; 2 Centro de Investigação em Saúde de Manhiça Manhica Mozambique; 3 Malaria Consortium London United Kingdom; 4 Malaria Consortium Maputo Mozambique; 5 National Institute of Health Maputo Mozambique; 6 GiveWell San Francisco, CA United States; 7 World Health Organization Maputo Mozambique; 8 IS Global Barcelona Spain; 9 The National Malaria Control Program Ministry of Health Maputo Mozambique

**Keywords:** malaria, chemoprevention, children, protocol, Nampula, Mozambique, feasibility, effectiveness, mixed methods, SMC, SP+AQ, hybrid effectiveness, cRCT

## Abstract

**Background:**

Seasonal malaria chemoprevention (SMC) is a highly effective community-based intervention to prevent malaria infections in areas where the malaria burden is high and transmission occurs mainly during the rainy season. In Africa, so far, SMC has been implemented in the Sahel region. Mozambique contributes 4% of the global malaria cases, and malaria is responsible for one-quarter of all deaths in the country. Based on recommendations in the Malaria Strategic Plan, the Malaria Consortium, in partnership with the National Malaria Control Programme in Mozambique, initiated a phased SMC implementation study in the northern province of Nampula. The first phase of this 2-year implementation study was conducted in 2020-2021 and focused on the feasibility and acceptability of SMC. The second phase will focus on demonstrating impact. This paper describes phase 2 of the implementation study.

**Objective:**

Specific objectives include the following: (1) to determine the effectiveness of SMC in terms of its reduction in incidence of malaria infection among children aged 3 to 59 months; (2) to determine the chemoprevention efficacy of sulfadoxine-pyrimethamine plus amodiaquine (SP+AQ) when used for SMC in Nampula Province, Mozambique, and the extent to which efficacy is impacted by drug resistance and drug concentrations; (3) to investigate the presence and change in SP+AQ– and piperaquine-resistance markers over time as a result of SMC implementation; and (4) to understand the impact of the SMC implementation model, determining the process and acceptability outcomes for the intervention.

**Methods:**

This type 2, hybrid, effectiveness-implementation study uses a convergent mixed methods approach. SMC will be implemented in four monthly cycles between December 2021 and March 2022 in four districts of Nampula Province. Phase 2 will include four components: (1) a cluster randomized controlled trial to establish confirmed malaria cases, (2) a prospective cohort to determine the chemoprevention efficacy of the antimalarials used for SMC and whether drug concentrations or resistance influence the duration of protection, (3) a resistance marker study in children aged 3 to 59 months to describe changes in resistance marker prevalence over time, and (4) a process evaluation to determine feasibility and acceptability of SMC.

**Results:**

Data collection began in mid-January 2022, and data analysis is expected to be completed by October 2022.

**Conclusions:**

This is the first effectiveness trial of SMC implemented in Mozambique. The findings from this trial will be crucial to policy change and program expansion to other suitable geographies outside of the Sahel. The chemoprevention efficacy cohort study is a unique opportunity to better understand SMC drug efficacy in this new SMC environment.

**Trial Registration:**

ClinicalTrials.gov NCT05186363; https://clinicaltrials.gov/ct2/show/NCT05186363

**International Registered Report Identifier (IRRID):**

DERR1-10.2196/36403

## Introduction

### Overview

The World Health Organization (WHO) defines seasonal malaria chemoprevention (SMC) as the intermittent administration of full treatment courses of an antimalarial medicine during the malaria season to prevent malarial illness, with the objective of maintaining therapeutic antimalarial drug concentrations in the blood throughout the period of greatest malarial risk [1-5]. It involves administering monthly courses of sulfadoxine-pyrimethamine (SP) and amodiaquine (AQ) during this peak transmission period to those most at risk: children 3 to 59 months of age [6]. SMC has been demonstrated to be a highly effective intervention that can prevent up to 75% of malaria cases in eligible children [7]. It has also been shown that the intervention can be delivered safely at scale, achieving high coverage [8,9]. To date, SMC has only been adopted and scaled up in Sahelian countries of West and Central Africa, following the WHO recommendation for this region to be the first to implement SMC. However, in many parts of East and Southern Africa, there remain concerns over widespread resistance to SP, which may reduce the effectiveness of SMC [10,11]. However, it has been suggested that SP may retain its protective efficacy even in areas where resistance is high [12,13].

Mozambique accounts for 4% of global malaria cases, and the disease is highly endemic in the entire country, with the highest prevalence in the north and along the coast [14-16]. According to surveys conducted in Mozambique, the national prevalence of malaria in children aged 6 to 59 months remained stable at 38% in 2011 [17], 40% in 2015 [18], and 39% in 2018 [19]. The prevalence varies across the country, with it being higher in the northern and central provinces and lower in the southern provinces. Nampula was one of the provinces with the highest prevalence of malaria (65%-66%) in 2015. However, a slight decrease was observed in 2018 to 48%, but it remained one of the highest prevalence rates after Cabo-Delgado (57%) [19]. Among chemoprevention strategies implemented in Mozambique, for more than 10 years, the Mozambique Ministry of Health and the National Malaria Control Programme (NMCP) have been actively implementing intermittent preventive treatment in pregnancy using SP [18].

To date, in Mozambique, studies about delivery of intermittent preventive treatment in infants (IPTi) with SP have been conducted and have been paired with the Expanded Programme on Immunization during routine contacts [20,21]. Even though different research studies about the resistance of SP in sub-Saharan Africa exist in the literature, there is no robust evidence about the impact of resistance on the efficacy of intermittent prevention treatments as an SMC strategy in the region [2,22] or IPTi in Mozambique [23]. A midterm review of the country’s Malaria Strategic Plan 2017-2022 recommended SMC as a strategy to accelerate the impact of treatment in the highest-burden locations [24]. To assess whether SMC can be an effective malaria prevention strategy in an area where resistance to SP is assumed to be high, the Malaria Consortium, in partnership with the NMCP in Mozambique, initiated a phased SMC implementation project in Nampula Province. The project was designed as a 2-year hybrid effectiveness-implementation study that took place over two consecutive phases.

### Phase 1

The first phase focused on exploring the feasibility and acceptability of SMC outside of the Sahel [25]. It was conducted between November 2020 and February 2021 and involved administering four monthly cycles of SMC to a target population of around 72,000 children in two districts of Nampula Province, Malema, and Mecubúri. SMC delivery followed the standard door-to-door delivery model commonly used in Sahelian countries, with trained volunteers acting as community distributors, supervised by health facility workers. A third district, Lalaua, where SMC was not implemented served as a control area. Research activities included the following:

1. Documentation of how the SMC implementation model used in the Sahel was adopted to the local context.
2. A representative end-of-round household survey to assess coverage and quality of delivery,
3. Interviews and focus group discussions with key stakeholders.
4. A quality assessment of health management information system (HMIS) data on malaria indicators reported at the health facility and district levels.
5. A nonrandomized controlled trial to estimate the ratio of the hazard of developing one or more episodes of malaria during SMC cycles among a sample of children in an intervention district compared to the hazard in the control district.
6. A study of molecular resistance markers to determine baseline prevalence of SP and AQ resistance and any increase in resistance prevalence after one annual round of SMC [25].

While data analysis is ongoing, preliminary results suggest that SMC is a feasible and acceptable intervention and that it confers protection from malaria to eligible children. Detailed results from phase 1 will be published elsewhere.

### Phase 2

The second phase will focus on demonstrating impact by addressing feasibility and effectiveness of SMC and chemoprevention efficacy. This protocol describes phase 2 of the implementation study.

### Objectives

Phase 2 aims to test the feasibility, effectiveness, and chemoprevention efficacy of SMC with SP plus AQ (SP+AQ) in Nampula Province in Mozambique, where malaria transmission is highly seasonal. Specific objectives include the following:

1. To determine the effectiveness of SMC in reducing the incidence of malaria infection among children aged 3 to 59 months.
2. To determine and measure efficacy as follows:
  - To determine chemoprevention efficacy of SP+AQ when used for SMC in Nampula Province, Mozambique.
  - To calculate the extent to which efficacy is impacted by drug resistance and drug concentrations.
3. To investigate the presence and change of SP+AQ– and piperaquine-resistance markers over time as a result of SMC implementation.
4. To understand the impact of the SMC implementation model by determining process, costing, and acceptability outcomes for the intervention.

## Methods

### Study Design

**Figure 1:**
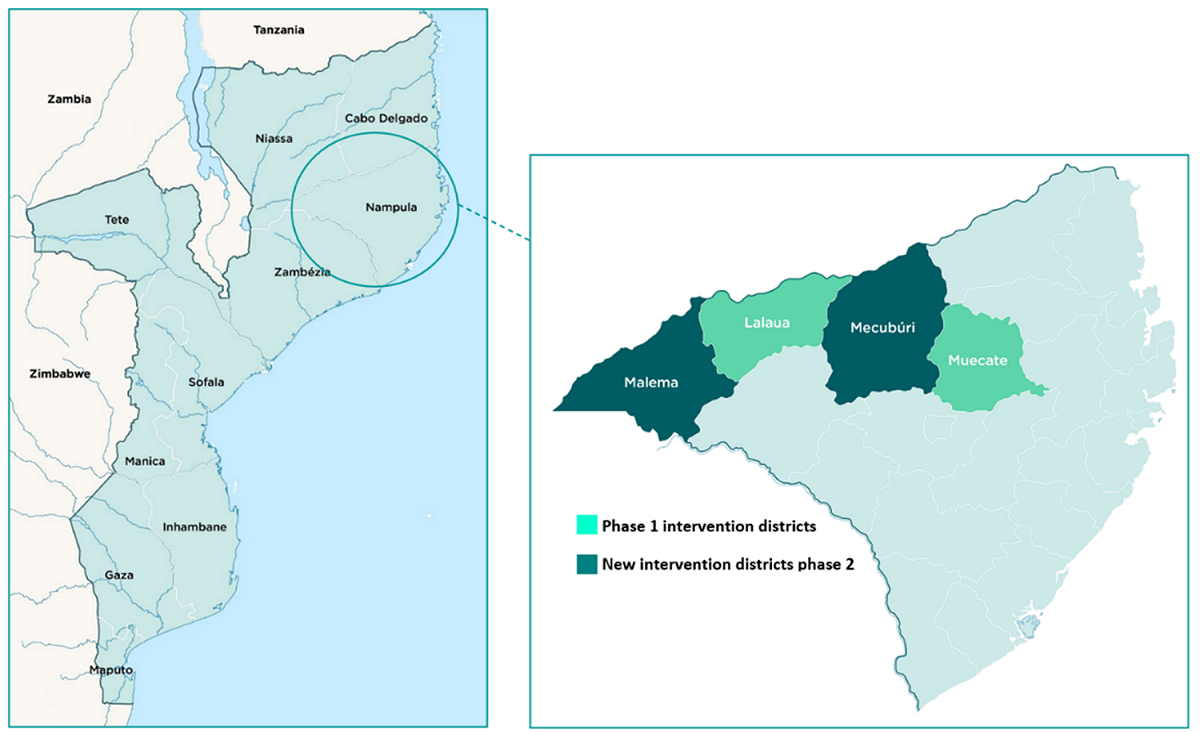
Intervention districts in Nampula Province, Mozambique.

This type 2, hybrid, effectiveness-implementation study uses a convergent mixed methods approach [26]. As the study includes components of clinical effectiveness and implementation research, a type 2, hybrid, effectiveness-implementation design was selected. These designs provide benefits over pursuing these lines of research independently; for example, they offer more rapid translational gains, more effective implementation strategies, and more useful information for decision makers. SMC will be implemented in four monthly cycles between January 2022 and April 2022 in four districts of Nampula Province (Figure 1): Malema and Mecubúri, where phase 1 of the study was conducted, will continue to receive SMC in phase 2; Lalaua, the control area in phase 1, and Muecate will receive SMC for the first time in phase 2. The research will involve the following components:

1. Conducting a cluster randomized controlled trial (cRCT) through passive surveillance to establish confirmed malaria cases among participating children.
2. Conducting a prospective chemoprevention efficacy cohort study to determine whether SP+AQ provides 28 days of protection from infection, and whether drug concentrations and resistance influence the duration of protection.
3. Conducting a resistance marker study in children aged 3 to 59 months in the two research districts plus the two standard intervention districts to describe changes in resistance marker prevalence over time.
4. Conducting a process evaluation of the SMC implementation looking at cost and process outcomes.

### Study Setting

Individual elements of the study will be conducted in all four districts. The cRCT and process evaluation will be conducted in Lalaua and Muecate districts in Nampula Province, Northeastern Mozambique (Figure 1). The chemoprevention efficacy cohort study will be conducted in communities within the catchment area of one health facility in Lalaua district. The resistance marker study will be conducted in all four districts. As part of the process evaluation, several key informant interviews may be conducted with stakeholders who are based in Maputo.

To identify suitable districts for SMC implementation, a suitability ranking was conducted in-country with oversight from the WHO for all provinces. Criteria included a variety of factors: (1) seasonality eligible for SMC (ie, 60% of rainfall concentrated into 4 months), (2) mortality (ie, areas of highest mortality in children younger than 5 years using HMIS data), (3) access to care (ie, highest ranking given to areas where access to care was lowest), and (4) treatment-seeking behavior (ie, highest ranking given to areas where treatment-seeking behavior was lowest). The average category score was used to determine a final ranking and identify the top 20 suitable districts for maximizing the impact of SMC. From the list of suitable districts, additional consideration was given based on the importance of implementing the intervention in an area where no other new interventions were taking place so that effects could be attributable to only the intervention under investigation. Districts in Nampula Province were selected, as no indoor residual spraying or new long-lasting insecticide-treated net distribution campaigns had been targeted to these areas, allowing for a more robust attribution of SMC impact on malaria. 

### Study Population

The study population that is eligible to receive SMC includes afebrile children [1] of either gender (ie, children with a fever on the day of SMC administration will be excluded and referred to the health facility for further evaluation), aged 3 to 59 months for any of the SMC cycles, and residing in any of the four districts. Children must be 59 months or younger at the start of the first cycle; if they turn 5 years old during the SMC season, they may continue to receive SMC. If children are 2 months old or younger at the first cycle month, they can begin SMC once they are 3 months old in any cycle. During phase 1, when only Mecubúri and Malema districts were included, population data were retrieved from the National Census 2017, adjusting by an annual growth factor of 3.5% in Nampula Province, as calculated by the National Institute of Statistics. For phase 2, population figures for Mecubúri and Malema were obtained through the administrative coverage data of SMC from phase 1. For the two new districts, Lalaua and Muecate, as it was done for districts in phase 1, population figures were retrieved from the 2017 census data, projected for 2021 and considering a growth rate of 3.5% per year (Table 1). Additionally, health workers involved in SMC implementation; caregivers of children younger than 10 years of age; community leaders and key stakeholders, such as health officials at different levels of the health system; and those involved in SMC implementation will be sampled to provide information with regard to the intervention, according to the different study elements.

**Table 1:** Estimated target population of seasonal malaria chemoprevention (SMC) districts.

District	Population^a^ for each eligible children’s group by age, n		
	3-11 months	12-59 months	All children
Malema	8500	35,800	44,300
Mecubúri	7300	36,000	43,300
Lalaua	3200	13,600	16,800
Muecate	1900	8000	9900
All districts	20,900	93,400	114,300

^a^District populations were determined by the National Institute of Statistics and SMC 2021 coverage data.

### Ethical Considerations

Ethical approval for this study was received from the Comité Nacional de Bioética para a Saùde (CNBS) of the Mozambique Ministry of Health on December 22, 2021 (reference No. 803/CNBS/21), and by the Research Ethics Committee of the Hospital Clinic of Barcelona on December 20, 2021 (reference No. HCB/2021/0944). Only participants who meet the inclusion criteria and whose caregivers provide written informed consent will be included in the study. The trial has been registered at ClinicalTrials.gov (NCT05186363).

### Primary and Secondary Outcomes

The primary outcomes for the different elements are presented below by component.

#### Cluster Randomized Controlled Trial

The primary outcome for this component of the protocol is the incidence of confirmed malaria cases using a rapid diagnostic test (RDT) reported through passive surveillance. Secondary outcomes include severe anemia, as an indication of severe malaria, and parasitemia levels, both gametocyte carriage rates and density of *Plasmodium falciparum* trophozoites and *P Falciparum* gametocytes.

#### Chemoprevention Efficacy Study

The primary outcome is chemoprevention failure in the presence of adequate drug concentration and associated parasite genotype, as defined by quantitative polymerase chain reaction (qPCR)–positive parasites on day 28 or a positive malaria slide at any time from day 7. Secondary outcomes include uncomplicated malaria within the first 28 days, hospitalization within the first 28 days, and severe malaria within the first 28 days.

#### Resistance Marker Study

The primary outcome is the prevalence of relevant SP+AQ– and piperaquine-associated antimalarial resistance phenotypes: *P falciparum* dihydrofolate reductase gene (*Pfdhfr*)—codons 51, 59, 108, and 164; *P* *falciparum* dihydropteorate synthetase gene (*Pfdhps*)—codons 431, 437, 540, 581, and 613; *P* *falciparum* chloroquine resistance transporter gene (*Pfcrt*)—codons 72 to 76; and *P* *falciparum* multidrug resistance gene 1 (*Pfmdr1*)—codons 86, 184, and 1246.

### Sample Sizes

#### Cluster Randomized Controlled Trial

Clusters will be selected for the intervention arm at the community level in two districts. The study will be powered to have an 80% chance of detecting a significant reduction in RDT-confirmed malaria incidence at the 5% confidence level using a chi-square test comparing two independent proportions in a cluster randomized design. We are aiming to detect a significant reduction in malaria incidence from 0.2 to 0.12 clinical episodes per child per high-transmission season, corresponding to the SMC round. The clinical episodes should be of sufficient severity to present to a health facility, and the reduction in episodes should be equivalent to an odds ratio of 0.66, or a minimum effect size of 33%. This is based on the assumption that the efficacy of SP+AQ in Northern Mozambique, where the prevalence of resistance alleles in circulating parasites is high, is half of that found in studies in West African settings. To account for the cluster randomized design, we assumed an intracluster correlation for malaria outcomes within communities of 0.23, based on data from SMC coverage surveys conducted by the Malaria Consortium in 2020 [27] and 2021 [28]. A cluster size of 15 children per community was selected, and children in the intervention and control arms were recruited in a ratio of 1:1.5. This resulted in a total sample size of 2850 eligible children in 190 clusters: 1710 in 114 clusters in the control arm and 1140 in 76 clusters in the intervention arm. A total of 76 communities from a list of all communities will be selected as intervention districts using a simple random method. Then, 114 communities will be selected at random from among those remaining for inclusion in the control arm.

#### Chemoprevention Efficacy Cohort Study

We aim to recruit participants from within Lalaua district, one of the new phase 2 implementation districts. Assuming a 3% breakthrough infection rate, sampling 494 children will provide a 95% CI of 1.7% to 4.9%. Assuming a 99% response rate and 1% loss to follow-up, a sample size of 500 children is considered appropriate. We assume that the drug resistance observed for all drugs (ie, SP+AQ) will be the same across a 300-km radius from the location. Therefore, selecting a single health facility should introduce no selection bias when we consider drug resistance prevalence across the region.

#### Resistance Marker Study

The sample size for the survey was determined using a sample size calculation from a WHO protocol for drug efficacy testing [29], with the intention to estimate changes in prevalence of SP- and AQ-resistance markers with enough precision to provide adequate power to detect changes if they occur. A sample of 300 children per district—150 each at baseline and end line—is expected to result in 90% power to detect a difference at the 5% confidence level in sextuple SP mutants from less than 1% to 2%. In Lalaua, where the chemoprevention efficacy study will be conducted, we determined that a sample of 600 children—300 each at baseline and end line—is needed to obtain a sufficiently accurate specific prevalence measure in the absence of data on malaria parasite ecologies. This will result in a total sample size across both districts of 1500, with 750 children sampled at both baseline and end line.

### Recruitment and Data Collection

#### Cluster Randomized Controlled Trial

In the control arm in both districts, compounds will be randomly sampled by researchers using household lists from selected communities, with one eligible child recruited at random from each household. After caregivers provide consent, children will be recruited and a short baseline questionnaire will be administered to collect individual data on each child and to confirm their eligibility. In the intervention arm, a researcher will follow a pair of community distributors as they administer SMC. In addition to variables such as experiences of fever within the past month among selected children, data on a range of variables relating to the selected child, their caregiver, and their household will be collected as part of the survey. These variables will include the selected children’s age, sex, and use of mosquito nets as well as the caregivers’ and heads-of-household’s level of education, occupational position, literacy level, household size, wealth (using the Simple Poverty Scorecard), primary language, and migration status, among other variables. Data on these variables will be collected in order to include potential confounders in the analysis for the association between the receipt of SP+AQ and clinically significant malaria. Children will be given SP+AQ by community distributors via directly observed therapy; in addition, blood samples will be taken and subsequent hemoglobin concentration measured using a HemoCue system (HemoCue AB). Children recruited into the study presenting at clinics will be identified using unique identification numbers and barcodes, and data on clinic visits, including suspected malaria cases and results of RDTs, will be matched to baseline questionnaire data to build a database for analysis.

#### Chemoprevention Efficacy Cohort Study

**Figure 2:**
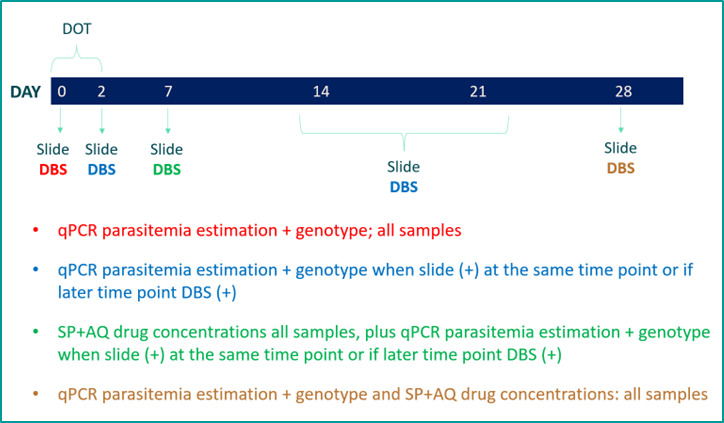
Proposed sample collection time points and subsequent sample processing sequence. DBS: dried blood spot; DOT: directly observed therapy; qPCR: quantitative polymerase chain reaction; SP+AQ: sulfadoxine-pyrimethamine plus amodiaquine.

This component will investigate the chemoprevention efficacy of SP+AQ at current dosing regimens among SMC-eligible children. Following the WHO’s Malaria Chemoprevention Efficacy Study Protocol [30], both malaria thick smears and dried blood spots (DBSs) will be taken at day 0, day 2, day 7, day 14, day 21, and day 28 during and after administration of the first cycle of SP+AQ, which will be administered through directly observed therapy. All DBS samples on day 0 and day 28 will be processed to ascertain low-density parasitemia estimates through qPCR. If sufficient DNA exists, day-0 and day-28 samples will be genotyped and compared. If children are found to have the same haplotype, this will indicate a recrudescent infection. If different haplotypes are present or if there is presence of an infection on day 28 that was not present on day 0, this will indicate a new infection after SP+AQ administration. Day-7 samples will also be processed for drug levels of all three SMC drugs, and day-28 samples will be processed for SP and AQ. The qPCR methodology is primed to detect presymptomatic parasitemia of clinical relevance. More sensitive PCR methodologies will not be conducted in order to prevent detection of extremely low–level parasitemia that will not be of clinical significance. DBSs will also be processed for estimates of blood parasite density and genotyping of relevant drug-resistance markers on days 2, 7, 14, and 21 if a child’s thick smear on those days was positive or if DBSs at a later time point are *P falciparum* positive. A DBS will also be collected whenever a child has a fever and is positive on a malaria RDT based on histidine-rich protein 2 and plasmodium lactate dehydrogenase. Drug level, qPCR parasite estimation, and genotyping will also take place. A blood smear will not be taken, as the RDT diagnosis will be sufficient when associated with fever. An RDT will not be given to children on day 0 unless they have signs and symptoms of malaria as per the standard SMC protocol, in which case they will be excluded from the study sample (see Figure 2 for an illustration of the proposed sample collection time points and subsequent sample processing sequence). We aim to recruit participants from within Lalaua, one of the new phase 2 research districts.

#### Resistance Marker Study

Trends in SP and AQ resistance will be monitored in the two original intervention districts, as well as in the two research districts. Additionally, in the new phase 2 areas, the prevalence of *P* *falciparum* plasmepsin 2 (*Pfplm2*) will be determined to inform future studies on appropriate use of dihydroartemisinin piperaquine for chemoprevention. A health facility–based, cross-sectional survey will be conducted before SMC project implementation (ie, baseline) and after one complete round of SMC distribution (ie, end line). Monitoring the prevalence of alleles associated with resistance to drugs is, by standard protocol, performed by collecting samples from symptomatic children with evidence of infection. The sample collection will be performed in four selected health facilities in each study district. The key markers to be monitored are as follows: *Pfdhfr*—codons 51, 59, 108, and 164; *Pfdhps*—codons 431, 437, 540, 581, and 613; *Pfcrt*—codons 72 to 76; *pfmdr1*—codons 86, 184, and 1246; and *Pfplm2*.

### Data Analysis

#### Cluster Randomized Controlled Trial

First, the characteristics of the eligible children in the intervention and control arms will be described and tabulated; these analyses will use survey weights obtained using population estimates for each community cluster. Next, a chi-square test will be performed to determine whether there is a significant difference in the proportions of children in each arm experiencing at least one confirmed malaria case during the study period, and exact odds ratios will be calculated.

Random-effects Cox proportional hazards regression models will then be fitted to estimate the difference in risk of an RDT-confirmed malaria case over time of follow-up; results will be expressed as hazard ratios, and a percentage effect size will be calculated. Model diagnostics will be run to verify the proportional hazards assumption, and random effects will be modeled at the community level to account for within-cluster correlation in malaria outcomes.

Two types of models will be fitted. The first will model risk of malaria based on time to first malaria case during each child’s first continuous period of follow-up; those experiencing an RDT-confirmed malaria case will be considered dropped from the sample, whereas those lost to follow-up will be considered right-censored. The second model type will account for recurrent events—using random effects for follow-up periods nested within individual children’s data—with children experiencing a malaria case considered to be “recovered” on the day of case confirmation and will continue to contribute follow-up information after this event.

Finally, to adjust for potential confounders, covariates based on data obtained from the baseline recruitment survey will be added sequentially to each of these two models by forward stepwise selection.

#### Chemoprevention Efficacy Cohort Study

Distributions and proportions of relevant mutations will be analyzed to compare the chemoprevention efficacy between groups of mutations. The blood drug concentrations will be analyzed as a cohort by mean, median, and SD, and statistical tests of their associations with treatment outcome—in particular, drug concentrations on day 7—will be performed. The focus will be on outliers with low levels of drug concentration based on the outcome measures described. Day-28 positivity will be associated with antimalarial drug resistance genotype.

#### Resistance Marker Study

Prevalence of *Pfdhps*, *Pfdhfr,* *Pfcrt*, and *Pfmdr1* alleles in parasites obtained from participating children will be ascertained. Copy numbers of *Pfplm2* will also be quantified.

### Availability of Data and Materials

The associated study protocol and data collection tools will be made available upon request from the corresponding author. Quantitative data sets are available from the corresponding author upon reasonable request after the completion of primary analyses and results dissemination. Qualitative study data sets will not be available, as they may include identifiable information that could comprise participant confidentiality.

## Results

Data collection began in mid-January 2022 and concluded in mid-June 2022. Data analysis is expected to be completed by October 2022.

## Discussion

### Principal Findings

This hybrid research protocol will provide information about the feasibility, effectiveness, and chemoprevention efficacy of SMC using SP+AQ in Nampula, a northern province of Mozambique, where malaria transmission is high and seasonal.

The results from phase 1 of the Mozambique SMC implementation study demonstrated that SMC with SP+AQ is safe, feasible, and acceptable in the local context. The intervention was successfully delivered according to schedule and at the anticipated scale, achieving high coverage [28]. No serious adverse events were reported. Acceptability of the intervention among the population was high, with no negative rumors reported. The intervention appears to be highly effective: in a nonrandomized controlled trial, children who lived in a district where SMC had been implemented had 86% lower odds of developing clinical malaria during the peak transmission season compared with children who lived in the control district without SMC implementation [31]. There were high rates of sulfadoxine and pyrimethamine resistance, but one annual round of SMC does not appear to have had a negative impact on the resistance profile.

In phase 2 of the study, more work is needed to understand the efficacy of SP+AQ to clear existing infections and prevent new infections. This is what we mean by the term “chemoprevention efficacy.” The phase 2 chemoprevention cohort will determine whether SP and AQ in combination using current dosing regimens for SMC-eligible children are able to prevent infections over a 28- to 31-day period between cycles during the high-transmission season. Importantly, this cohort will also determine whether these drugs can also clear asymptomatic malaria to provide an estimate of chemoprevention efficacy in terms of prevention of both new and recrudescent infections. This will help determine the duration of effectiveness of SP+AQ. Further work is also crucially needed to map antimalarial drug resistance in these key populations in Mozambique, which has already been frequently highlighted in the literature [32]. The relationship between chemoprevention effectiveness and chemoprevention efficacy and how it relates to drug resistance is currently unclear. The chemoprevention efficacy component of this study aims to elucidate that relationship within the context of Northern Mozambique. The intention is to create a system where the relationship between these variables is better understood within and across malaria ecologies. We may find that drug resistance across Northern Mozambique and how it relates to effectiveness and efficacy covers a large geographical area, but this must be confirmed through future work once we better understand the dynamics of these interactions. Therefore, in the process of validating this methodology and analyzing these dynamics, it is prudent to create a geographical restriction to the assumptions of this protocol, whereby resistance to SP, in particular, may be representative of a given parasite population [13].

The study will also provide robust evidence of the effectiveness of SMC in an area outside of the Sahel in the form of a randomized controlled trial. This is the first time this work will have been done outside the Sahel and will prove crucial to national and global policy change. This study will support the extension of SMC outside Sahel geographies as already outlined by the WHO [33]. Work in close collaboration with key stakeholders, including the NMCP, the Mozambique Ministry of Health, and the Mozambique National Institute of Health, will ensure that clear and consistent recommendations will be provided in order to produce robust guidance for the national malaria control strategy. Findings will be presented at the province and district levels during meetings led by the research team and local stakeholders; these will involve community leaders, community distributors, and health professionals. Key messages will be provided to the audience, who will have the opportunity to give feedback regarding the development of new actions to secure continuity. Findings will also be presented during the next Mozambique Regional Scientific Days edition in 2023. Finally, results will be disseminated in peer-reviewed scientific journals and on the official Malaria Consortium website.

### Limitations

Administration of SP+AQ to ineligible children older than 59 months may raise concerns in relation to the development of drug resistance, as doses administered are unlikely to offer sufficient protection against malaria transmission. This also influences the secondary data analysis in the same way; administering SP+AQ to children older than 59 months could reduce the apparent effect size in the targeted age group.

## Abbreviations

AQ: amodiaquine
CNBS: Comité Nacional de Bioética para a Saùde
cRCT: cluster randomized controlled trial
DBS: dried blood spot
HMIS: health management information system
IPTi: intermittent preventive treatment in infants
NMCP: National Malaria Control Programme
*Pfcrt*: *Plasmodium falciparum* chloroquine resistance transporter gene
*Pfdhfr*: *Plasmodium falciparum* dihydrofolate reductase
*Pfdhps*: *Plasmodium falciparum* dihydropteorate synthetase
*Pfmdr1*: *Plasmodium falciparum* multidrug resistance gene 1
*Pfplm2*: *Plasmodium falciparum* plasmepsin 2
qPCR: quantitative polymerase chain reaction
RDT: rapid diagnostic test
SMC: seasonal malaria chemoprevention
SP: sulfadoxine-pyrimethamine
SP+AQ: sulfadoxine-pyrimethamine plus amodiaquine
WHO: World Health Organization
